# Utilizing Chinese Admission Records for MACE Prediction of Acute Coronary Syndrome

**DOI:** 10.3390/ijerph13090912

**Published:** 2016-09-13

**Authors:** Danqing Hu, Zhengxing Huang, Tak-Ming Chan, Wei Dong, Xudong Lu, Huilong Duan

**Affiliations:** 1 College of Biomedical Engineering and Instrument Science, Zhejiang University, Hangzhou 310027, China; danqinghu1990@zju.edu.cn (D.H.); lvxd@zju.edu.cn (X.L.); duanhl@zju.edu.cn (H.D.); 2 Philips Research China—Healthcare, Shanghai 200233, China; Cyrus.chan@philips.com; 3 Department of Cardiology, Chinese PLA General Hospital, Beijing 100853, China; 301dongw@sina.com

**Keywords:** MACE prediction, acute coronary syndrome, admission record, hybrid model, risk factor identification

## Abstract

*Background*: Clinical major adverse cardiovascular event (MACE) prediction of acute coronary syndrome (ACS) is important for a number of applications including physician decision support, quality of care assessment, and efficient healthcare service delivery on ACS patients. Admission records, as typical media to contain clinical information of patients at the early stage of their hospitalizations, provide significant potential to be explored for MACE prediction in a proactive manner. *Methods*: We propose a hybrid approach for MACE prediction by utilizing a large volume of admission records. Firstly, both a rule-based medical language processing method and a machine learning method (i.e., Conditional Random Fields (CRFs)) are developed to extract essential patient features from unstructured admission records. After that, state-of-the-art supervised machine learning algorithms are applied to construct MACE prediction models from data. *Results*: We comparatively evaluate the performance of the proposed approach on a real clinical dataset consisting of 2930 ACS patient samples collected from a Chinese hospital. Our best model achieved 72% AUC in MACE prediction. In comparison of the performance between our models and two well-known ACS risk score tools, i.e., GRACE and TIMI, our learned models obtain better performances with a significant margin. *Conclusions*: Experimental results reveal that our approach can obtain competitive performance in MACE prediction. The comparison of classifiers indicates the proposed approach has a competitive generality with datasets extracted by different feature extraction methods. Furthermore, our MACE prediction model obtained a significant improvement by comparison with both GRACE and TIMI. It indicates that using admission records can effectively provide MACE prediction service for ACS patients at the early stage of their hospitalizations.

## 1. Introduction

Acute coronary syndrome (ACS) refers to a group of conditions due to decreased blood flow in the coronary arteries such that part of the heart muscle is unable to function properly or dies [1,2]. Major adverse cardiovascular events (MACE) is used to denote the composite of a variety of adverse events related to the cardiovascular system [3,4]. Clinical MACE prediction of ACS, as an important and widely studied topic, has significant impact on medical decision making for ACS patient care and treatment, and clinical outcome evaluation [1,5]. Extensive research efforts have been devoted to MACE prediction of ACS patients. Traditionally, many ACS risk scoring tools, e.g., GRACE [6], TIMI [7], Pursuit [8], and Framingham [9], etc., are developed based on population samples monitoring over a long period of time [10]. Relied on a pre-selected set of risk factors identified in these models, the goal of these ACS risk scoring tools was to predict the risks of patients [11]. Although useful, these tools have several important weaknesses, e.g., they only consider a limited number of risk factors, have difficulty in coping with missing risk factors, and required the selection of a standard tool to be applied in clinical practice, etc. [5].

**Figure 1:**
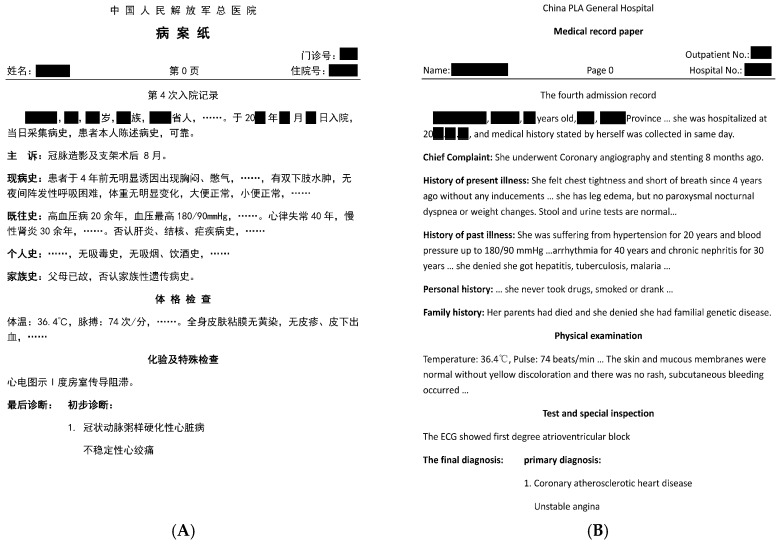
An example of part of an admission record collected from a Chinese hospital. (**A**) Original admission record in Chinese; and (**B**) its translation into English.

Recently, with the rapid development of hospital information systems, a large volume of electronic health records (EHRs) has become available, which offers the opportunity to alleviate the aforementioned limitations for MACE prediction of ACS patients [12,13,14]. In particular, admission records, as an important type of EHRs, contain a wealth of information to describe patient conditions at the early stage of length of stay (LOS), and thus can be utilized for MACE prediction in a proactive manner [13]. Figure 1 shows a de-identified sample of admission records collected from a Chinese hospital. It contains valuable patient information like demographics, medical history, physical examination results, lab test and specific inspection, etc. In addition, the first primary diagnosis and common comorbidities of the patient are given at the end of the admission record. In clinical practice, physicians often refer to the admission record of an ACS patient to determine his/her clinical risk. In this sense, it is possible to utilize admission records, study medical cases, extract significant ACS risk factors, and exploit these for helping physicians predict MACE for patients at the early stage of their hospitalizations, so as to make the practice better for the care of individual patients [15,16,17].

In this study, we propose a hybrid approach by utilizing admission records for MACE prediction. Specifically, we firstly extracted structured patient features from a large volume of admission records. Two well-known methods, namely the rule-based medical language processing (RBMLP), and Conditional Random Fields (CRFs), were assessed to show that essential patient features can be consistently extracted from a large volume of admission records. After that, we applied several representative supervised learning algorithms, namely Support Vector Machine (SVM), Random Forest (RF), Naïve Bayes (NB) and Logistic Regression with ℓ_1_ regularization on the regression coefficients (ℓ_1_-LR), to construct MACE prediction models, and comparatively evaluated the performances of the learned models on a real clinical dataset consisting of 2930 patient samples, which were collected from the cardiology department of Chinese PLA General Hospital.

The remainder of this paper is organized as follows: Section 2 summarizes some related works. Section 3 describes the proposed approach for MACE prediction using admission records. Section 4 illustrates our experiment results on a clinical dataset collected from a Chinese hospital. In Section 5, we discuss the limitations and some future works in our study. Finally, some conclusions are given in Section 6.

## 2. Related Work

As a crucial problem for medical informatics, risk prediction is necessary to modern clinical decision support systems by providing healthcare practitioners an assessment of an individual’s risk against an adverse outcome [13,18]. Recent years, a lot of studies have focused on this topic. For example, Fonarow et al. [19], developed a classification and regression tree using CART for risk prediction of patients hospitalized with Acute Decompensated Heart Failure (ADHF), the experiment results indicated the risk of in-hospital mortality can be reliably estimated using routinely available vital signs and laboratory data obtained in hospital admission. Karaolis et al. [20], employed a C4.5 algorithm with five different splitting criteria methods to assess the risk factors of coronary heart events, which included myocardial infarction (MI), percutaneous coronary intervention (PCI) and coronary artery bypass graft surgery (CABG). Besides the events prediction models, they also obtained three rules with three risk factors, i.e., smoking, blood pressure and cholesterol, which can be controlled. Dong et al. [12], proposed a new genetic fuzzy algorithm to develop an unstable angina assessment model. Physicians manually evaluated the patients’ risk to support fuzzy association rules mining for model construction. Liu et al. [21], proposed an ensemble-based scoring system (ESS) to predict acute cardiac complications within 72 h based on heart rate variability, 12-lead ECG and vital signs data. In particular, they adopted a hybrid-sampling approach to manipulate the imbalanced data in their study. 

With the widely adoption of EHRs in healthcare organizations, a large number of studies have explored the utilization of EHRs for risk prediction and more advanced machine learning algorithms were introduced into this topic. Singh et al. [22], employed a novel logistic regression with ℓ_2_ regularization on the regression coefficients (ℓ_2_-LR) to develop predictive models incorporating temporal EHR data. Their experiment results indicate that exploiting temporal information can yield improvements in predicting deterioration of kidney function. Churpek et al. [23], employed a person-time multinomial logistic regression to develop a prediction model for adverse events, i.e., cardiac arrest and intensive care unit transfer, using EHR data to overcome the vital sign-based risk scores’ limited accuracy. The results indicate including laboratory values in the prediction model contributes important knowledge to the field. Bandyopadhyay et al. [14], presented a machine learning approach based on Bayesian network trained on electronic health data (EHD) to predict the probability of having a cardiovascular events, which can overcome the missing data and censored outcome data issues. Besides of the structured EHRs, the various unstructured clinical texts have been employed to supplement the prediction task. Pineda et al. [24], compared the machine learning classifiers for influenza detection from emergency department free-text reports. They employed a pipeline-based NLP system, namely Topaz, to extract 31 features from the reports and developed the classifiers with three different missing value strategies. The results show machine learning classifier achieved a better performance than an expert-constructed classifier, and missing value and imbalance data issues have little influence on the prediction task. Jonnagaddala et al. [14], developed a method to calculate the Framingham Risk Score from unstructured EHRs using clinical text mining. They extracted the eight risk factors used in Framingham from the unstructured EHRs collected from the i2b2 2014 shared task 2 and employed a hybrid imputation strategies to handle the missing values in the extraction results.

However, as can be easily pointed out, nearly all the aforementioned studies have been estimated using a small hand-picked subset of features from highly stratified patient cohorts and developed their models using structured data. To the best of our knowledge, there have not been any studies for MACE prediction of ACS patients using unstructured admission records. Motivated by these observations, in our study, we proposed a hybrid approach by extracting as many informative features as possible from the free-text admission records without any cohort inclusion criteria, in which offers a novel and complementary approach for MACE prediction of ACS patients.

## 3. Methods

**Figure 2:**
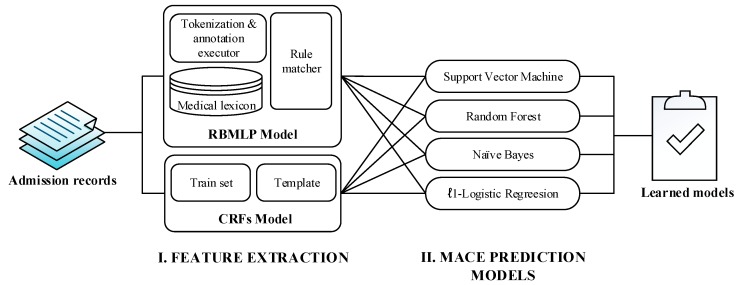
The pipeline of the proposed approach for MACE prediction.

In this study, we built a hybrid approach for MACE prediction, which leverages patient features extracted from admission records. The pipeline of the proposed approach is depicted in Figure 2, which consists of several functional components described in the following subsections. In brief, we firstly extracted patient features from admission records using two popular feature extraction methods, namely the rule-based medical language processing (RBMLP) and Conditional Random Fields (CRFs). After that, we employed four well-established supervised learning algorithms to build our MACE prediction models.

### 3.1. Feature Extraction

In our study, we aimed to construct the dataset from admission records to support the MACE prediction. So a crucial procedure is feature extraction from the free texts of admission records, which plays the key role to describe the patient conditions at their early stages of hospitalization. Here one meaningful question to explore beforehand is how sensitive the machine learning results are with respect to a different feature extraction methods [24]. To assess that, we employed two popular feature extraction methods to process the free text of admission records, one is the Rule-Based MLP method (RBMLP) and the other is a machine learning method, namely Conditional Random Fields (CRFs).

#### 3.1.1. Rule-Based Medical Language Processing Method

As shown in Figure 1, the patient information is narrated in a relatively regular manner in Chinese in the admission record. For example, if there is a stenosis appeared in a vessel, the descriptive word “stenosis” always locates behind the word “vessel” and all related information is described in a clause separated by comma. According to this fact, we developed a RBMLP model to extract essential patient features from admission records. Our RBMLP model consists of three core modules, i.e., medical lexicon, tokenization and annotation executor, and rule matcher:

- A comprehensive medical lexicon which supports the automatic lexical tagging process is indispensable [25,26]. In this study, we manually extracted and encoded lexemes and their semantic classes in the clinical pathway specification published by the host hospital and merged them into the medical lexicon described in [27] as our prototype lexicon. To improve the prototype lexicon, we first removed the lexemes in some sematic classes, i.e., Unit, Number, Time and etc., we didn’t need in our methods, and then we mapped the lexicon to 50 admission records to added the missing critical lexemes, i.e., missing descriptive lexemes, symptom and diagnosis lexemes in different forms from the one in lexicon, and etc., to our medical lexicon. We repeatedly consulted clinicians to clarify the lexemes and validate the results during the lexicon construction. Totally 230,284 lexemes were included in the lexicon which covered 15 semantic classes.
- Supported by the lexicon mentioned above, the tokenization module was implemented based on a reverse directional maximum match method [28], which segmented the text into words or phrases, and semantic classes were annotated by special symbols in the meantime. The symbol annotated to support rule matching was constructed by a semantic class abbreviation (“ST” is the abbreviation of “Symptom”) to indicate the word or phrase’s semantic class, a special character structure (we used “#” as our structure) to distinguish the symbol from text and an index to retrieve the original word or phrase of the symbol. We used the symbols to replace the original words or phrases to support the process of rule matcher. Four pairs of original and annotated clauses can be found in Table 1.
- By observing 50 training admission records, we identified 18 rules to match the information. Rule matcher was executed in the following steps. Firstly, each annotated admission record was decomposed into clauses based on a punctuation-driven sentence boundary detection algorithm [29]. Then all annotated clauses were matched sequentially by a total of 18 rules defined in the rule matcher. A negation detection algorithm was applied to determine if matched concepts were affirmed or negated. Four representative rules were used for matching clauses, as shown in Table 1.

#### 3.1.2. Conditional Random Fields Method

To evaluate whether different feature extraction methods can influence the results of risk prediction models significantly, we employed another feature extraction method, i.e., Conditional Random Fields (CRFs), to extract patient features again. CRFs is a framework for building probabilistic models to segment and label sequence data [30]. This state-of-the-art machine learning model is widely used on named entity recognition, text chunking and especially information extraction. In our work, we manually remarked what we want to extract in the training set with BIO tags, where “B” indicates a character is the beginning of the feature, “I” indicates a character is in the feature, and “O” indicates a character is out of the feature [25]. A total of 100 admission records were randomly selected and remarked as the training set to support the CRFs model construction.

To construct the features template, we used basic context features such as C_n_ and C_n_/C_n+1_ where C_0_ represents the current Chinese character and C_n_ represents the n^st^ character from the current character, and two binary features P(C_n_) and N(C_n_) indicate whether the current character is a punctuation (e.g., “，”) or a negative word (e.g., “未”), respectively. Details of our template are shown in Table 2. We employed CRF++ [31] to train our model and extracted the characters tagged “B” and “I” continuously as the output feature and a negation detection algorithm was applied in the meantime.

### 3.2. MACE Prediction Model

Based on steps above, we can extract a set of patient features from admission records to describe the patient conditions at their early stage of hospitalization. And then we can build our MACE prediction models using well-established machine learning algorithms with their predictive accuracy on the hold-out samples. In this study, 4 representative supervised machine learning classifiers, including Support Vector Machine (SVM), Random Forest (RF), Naïve Bayes (NB) and Logistic Regression with ℓ1 regularization on the regression coefficients (ℓ1-LR), were selected and compared to each other due to their demonstrated predictive performance and their popularity in the recently published research literature. All classifiers were binary classification models and 5-fold cross validation was performed on each classifier. We repeated the learning process 10 times to measure the average performance and the 95% confidence interval of each classifier. Below is a brief introduction to the employed algorithms as clinical risk prediction tools:

*SVM* is one of the most popular classification models based on constructing a hyperplane or set of hyperplanes in a high- or infinite-dimensional space to classify [32]. SVM works by mapping data to a high dimensional feature space. The mapping functions can be either a classification or regression function. There are four kernel functions, i.e., linear function, polynomial function, radial based function (RBF), and the sigmoid function, to be used in classification problems when the input data are not easily separable. In this study, we used the RBF kernel to build the non-linear SVM classifier with parameters determined by cross validation [33].

*RF* is an ensemble learning method that operates by constructing a multitude of decision trees and outputting the class that is the mode of the classes of the individual trees [34]. To classify a new object from an input vector, put the input vector down each of the trees in the forest. Each tree gives a classification, and “votes” for that class. The forest chooses the classification having the most votes. In this study, we constructed the RF classifier by setting 1000 as the number of trees in the forest and the square root of the number of features as the number of tried at each split [34].

*NB* is a simple probabilistic model based on Bayes’ theorem that assumes there is strong independence between the features [35]. This helps alleviate problems stemming from the curse of dimensionality, such as the need for feature sets that scale exponentially with the number of features.

*ℓ1-LR* is the LR with a lasso penalty factors on the regression coefficients [36]. *ℓ1-LR* has been widely used for many classification problems, particularly ones with many features. It is well-known that regularization is required to avoid over-fitting, especially when there are a large number of parameters to be learned. Hence, *ℓ1-LR* can be used for feature selection, and has been shown to have good generalization performance in the presence of many irrelevant features [36]. The admission records often contain a large volume of patient features, and many of them may be irrelevant to the problem of MACE prediction. In this sense, *ℓ1-LR* is appropriate for MACE prediction with many patient features extracted from admission records. 

Instead of providing a detailed description of these algorithms, we focus on reviewing the selected algorithms with the purpose of discussing important issues in a fundamental comparative analysis. To this end, we used two well-known performance measures, i.e., the receiver operating characteristic (ROC) curve and the area under the ROC curve (AUC), to evaluate the learning algorithms. A nonparametric method developed by DeLong et al. [37], which is common method used in biomedical research, was employed to compare the models’ curves to explore the differences between them. All model constructions and statistical analyses were completed using R version 3.2.1 (The R Foundation for Statistical Computing, Vienna, Austria).

## 4. Experiments and Results

### 4.1. Data

A collection of electronic health records (EHRs) consisting of 3463 ACS patient samples was obtained from the Cardiology Department of the China PLA General Hospital. The EHRs cover heterogeneous aspects like admission records, progress notes, radiological examination reports, etc., and thus can be used to provide MACE prediction service at an early stage of hospitalizations. We excluded the patient samples with incomplete admission records and progress notes from the dataset. Finally, a total of 2930 patient samples were included to develop our MACE prediction model. The average LOS of selected patients is 8.20 days, while some patients took a very short time, e.g., only 1 day in hospital, and others much longer, e.g., more than 3 months in hospital. It implicitly indicates the diversity of patient conditions in treatment processes.

**Figure 3:**
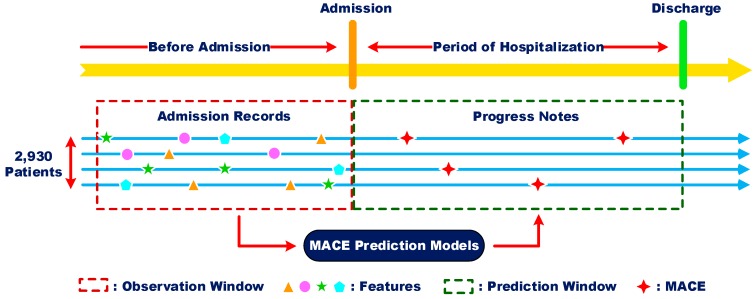
The temporal relationship of data for MACE prediction.

In this study, we extracted patient’s features from admission records mainly based on the two NLP techniques, i.e., RBMLP and CRFs, to construct MACE prediction models. An admission record sample has been shown in Figure 1. Furthermore, to determine whether the patients got MACE in their hospitalization, we recruited 4 clinical engineers to manually annotate the ischemic MACE, including all-cause death, Myocardial infarction, Angina attack, Heart failure, Arrhythmias, Re-revascularization, Transfer to undergo the CABG, Long length of stay, found in patient progress notes. Each progress note was annotated by at least three clinicians and the final results were determined over majority voting criterion by the three clinicians’ annotation results. The temporal relationship of data is illustrated in Figure 3, which indicates we employed the admission records to predict the MACE occurred in patients’ hospitalization. 

In future applications, MACE annotations can be theoretically extracted in an automatic manner leveraging natural language processing (NLP) technology. However, as a proof-of-concept study here, evaluating NLP annotations is beyond our focus and complicates the assessment of the machine-learned MACE prediction models. Therefore, in our experiment evaluation, we used manual annotations to eliminate this level of complexity. Finally, we obtained 752 patient samples (752/2930) with MACE in their hospitalization and the remaining 2178 (2178/2930) without MACE were as control group. Summary statistics of 2930 patient samples were illustrated in Table 3.

The case study was performed in the Cardiology Department at the Chinese PLA General Hospital. Prior approval was obtained from the data protection committee of the hospital to conduct the study (No. H1814). We state that the patient data were anonymized in this study.

### 4.2. Feature Extraction and Evaluation

#### 4.2.1. Feature Extraction Results

To explore how sensitive the machine learning results are with respect to different feature extraction methods, we employed two methods, one is a RBMLP method and the other is a CRFs method, to extract the features from the free texts of admission records, respectively. Note that patient’s vital signs and lab test results were recorded in a structured manner, so we only needed to employ simple regular expressions to extract these features. Summary statistics of dataset are shown in Table 4 and Table 5. 

Table 4 shows the numbers of feature types categorized into different classes. Table 5 lists the vital sign-related features’ means and standard deviations and the top 10 in lab test-related and free text features with most abnormalities are shown.

#### 4.2.2. Feature Extraction Evaluation

To evaluate the performances of the proposed feature extraction methods, we randomly selected 100 testing patient samples from the pool of admission records excluding the 50 records used in the lexicon supplement and rule identification and 100 records used in the CRFs training set. An experienced clinician was recruited to annotate the features of the testing set. Finally, a total of 4177 and 3897 feature-value pairs were annotated by the clinician for RBMLP and CRFs method, respectively.

We computed the standard metrics of precision, recall and F1 score for each method. For the RBMLP method, a total of 4037 feature-value pairs were extracted, in which 3979 were equal to the manually annotated records and 58 were not. Thus, the precision, recall and F1 score of the rule-based MLP method were 98.56%, 95.26% and 96.88%. For the CRFs method, a total of 3754 feature-value pairs were extracted, in which 3685 were same and 69 were inconsistent with the manually annotated patient records. Thus, the precision, recall and F1 score were 98.15%, 94.56% and 96.33%. As a result, both feature selection methods achieved consistently high accuracy compared to manual annotations.

To further analyze the false negative and false positive samples, we found there were two main reasons for incorrect feature extraction. One reason is text errors in admission records, e.g., punctuation misuse and misspellings. The other reason is that the rules we identified cannot cover all possible scenarios narrated in admission records. Note that, in our study, we focus on the method that utilizes the admission records for MACE prediction but not the feature extraction task. The evaluation results indicate the features extracted by the two methods are quite reliable and can effectively reflect the patient conditions such that they are suitable for our prediction task.

### 4.3. MACE Prediction Model Construction and Evaluation

To construct our models, we employed four representative supervised classification methods (i.e., SVM, RF, NB, and ℓ1-LR) mentioned in Section 3.2. In comparison with our machine-learned models, the well-known ACS risk score tool, i.e., GRACE (which was established by the full spectrum of ACS patients (ST-segment elevation myocardial infarction, non-ST-elevation myocardial infarction and unstable angina) and has shown excellent ability to assess the patient’s in-hospital risk [38]), and the TIMI risk score (which was established based on composite end point included all-cause mortality, new or recurrent MI, or severe recurrent ischemia requiring urgent revascularization), were employed as the baseline approaches to predict the MACE in our dataset. We employed metrics of the receiver operating characteristic curve (ROC) and the area under the curve (AUC) to evaluate the learned models and the baseline models. Table 6 shows the AUC values with 95% CI by the 10 times repetition of learning process using 5-fold cross validation. As shown in the table, all four models have shown good performances based on the datasets constructed by both RBMLP and CRFs methods. Random Forest achieved the best performance with 0.72 AUC value.

Furthermore, the differences between our models and the baseline models were assessed by the nonparametric method developed by DeLong et al. [37] and shown in Table 7. According to the comparison results, our learned models achieve better performances with a significant margin in comparison with the baseline models (Cells: E1–E4 and K7–K10 for GRACE; Cells: F1–F4 and L7–L10 for TIMI), which indicated a significant difference between our models and the baseline models. Moreover, the Random Forest obtains a significant improvement in comparison with Naïve Bayes (Cells: C1, I1, G3, I7), and relatively obvious improvements with ℓ_1_-Logistic Regression (Cells: D1, J1, G4, J7) and Support Vector Machine (Cells: B1, H1, G2, H7).

**Figure 4:**
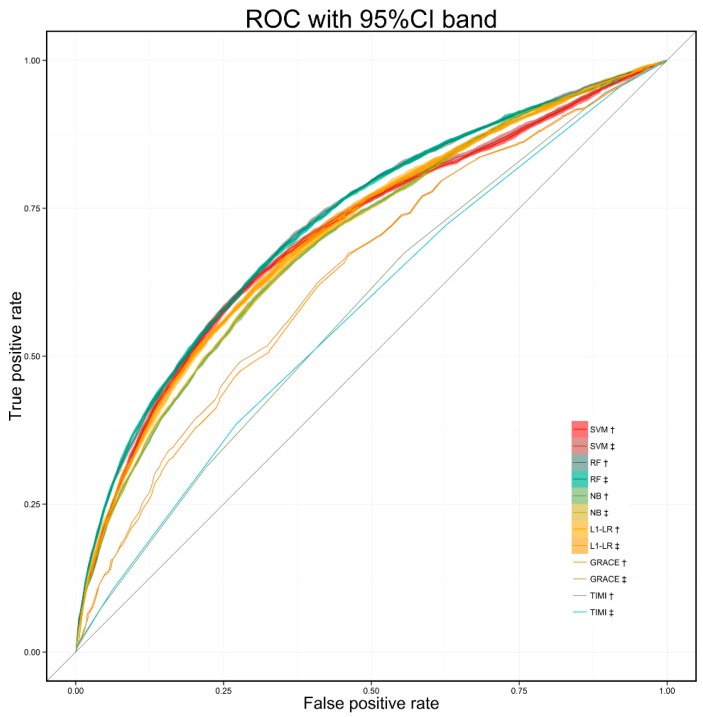
Averaged ROC with 95% CI band over 10-times repetition (†: RBMLP method; ‡: CRFs method).

**Figure 5:**
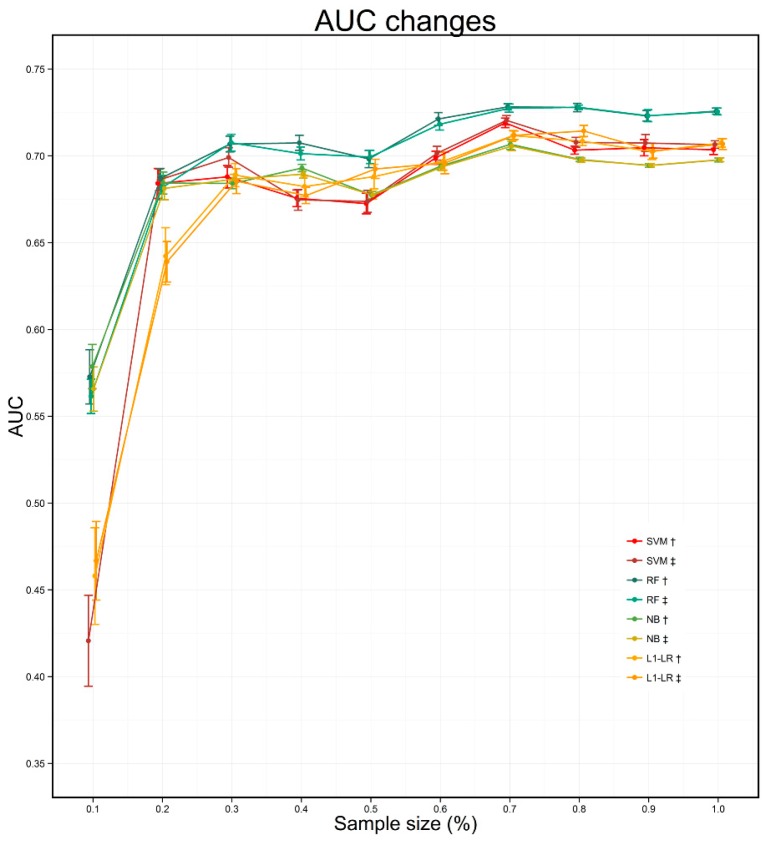
AUC changes under different sample size (†: RBMLP method; ‡: CRFs method).

In Figure 4 and Figure 5, we present a more comprehensive comparison between the four learned models and the baseline models. As shown in Figure 4, for the averaged ROC over 10-time repetition, all our learned models outperform the baseline models by a considerable margin in MACE prediction and there is little difference between the four learned models’ performances and confident intervals. Moreover, the influences of changes of sample sizes on AUC values were illustrated in Figure 5. We randomly selected the same proportion of samples from positive and negative samples to construct datasets in different sizes. The AUC values of all four models gradually ascend in overall trend along with the increases of the sample sizes. And all models were able to achieve relatively stable performances with 20% of all patient samples, which illustrates that our models can obtain good performances under a small sample size.

By comparing the performances of models built with datasets constructed by the RBMLP and CRFs methods in Figure 4 and Figure 5, Table 6 (Cells: G1, H2, I3, J4, K5, L6), it is easy to see that there are few differences in ROC curves and AUC values between the models’ performances, although the results were obtained based on different methods, which illustrates that our approach has a competitive generality regardless of the employed feature extraction methods.

Depending on the evaluation of the MACE prediction models illustrated above, we can conclude that our learned models achieve better performances than the traditional risk score models. However, one natural question is, if this improvement is mainly caused by the extracted features or the employed machine learning algorithms. To answer this question, we employed the features included in GRACE. As shown in which has a better performance than TIMI, and the four machine learning algorithms mentioned above to train models called models-GRACE.

Table 8 shows the AUC value of each model-GRACE and Table 9 shows the differences between GRACE, models-GRACE and our learned models. As illustrated in Table 8 and Table 9, we can notice that the SVM-GRACE models don’t obtain any improvement compared with GRACE in both RBMLP and CRFs datasets but gain significant improvement when using all extracted features. The RF-GRACE models obtain continuous improvement from GRACE to models-GRACE to learned models. However, the NB-GRACE and ℓ1-LR-GRACE models show different characteristics, which they obtain significant improvement when just employing features in GRACE but gain no further elevation in AUC when using all extracted features.

### 4.4. Potential Risk Factors

**Figure 6:**
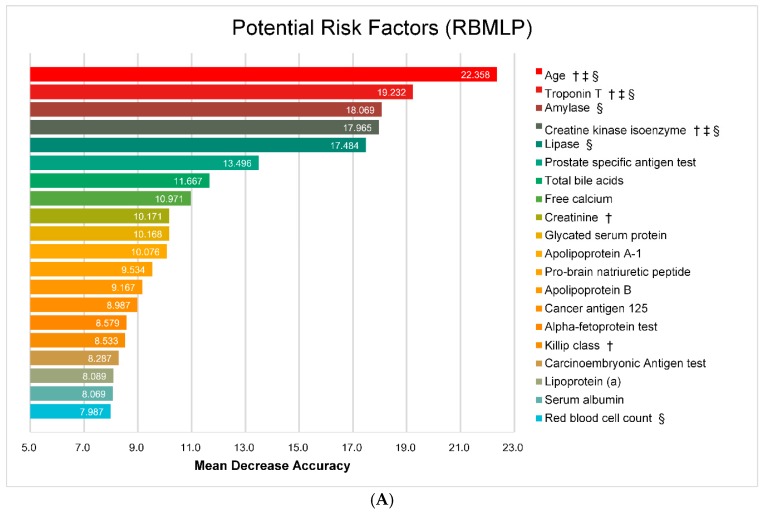

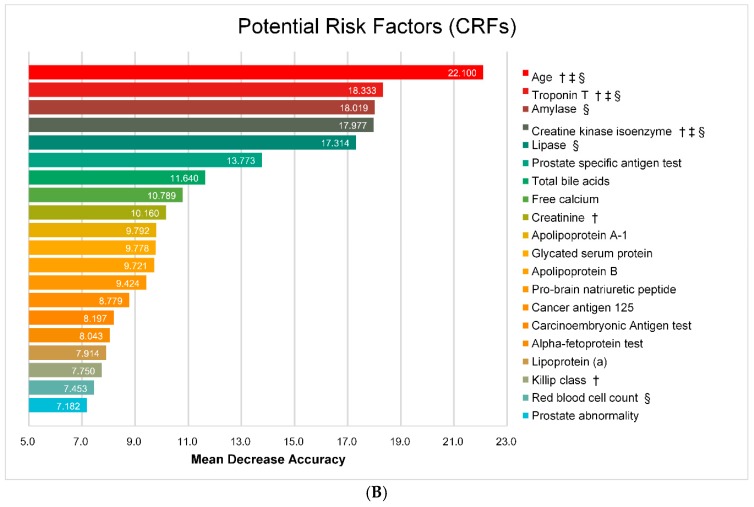
Potential risk factors based on random forest models. (**A**) Risk factors based on RBMLP; (**B**) Risk factors based on CRFs (†: risk factor employed in GRACE; ‡: risk factor employed in TIMI; §: risk factor employed in top 20 of both random forest and ℓ1-LR model).

**Figure 7:**
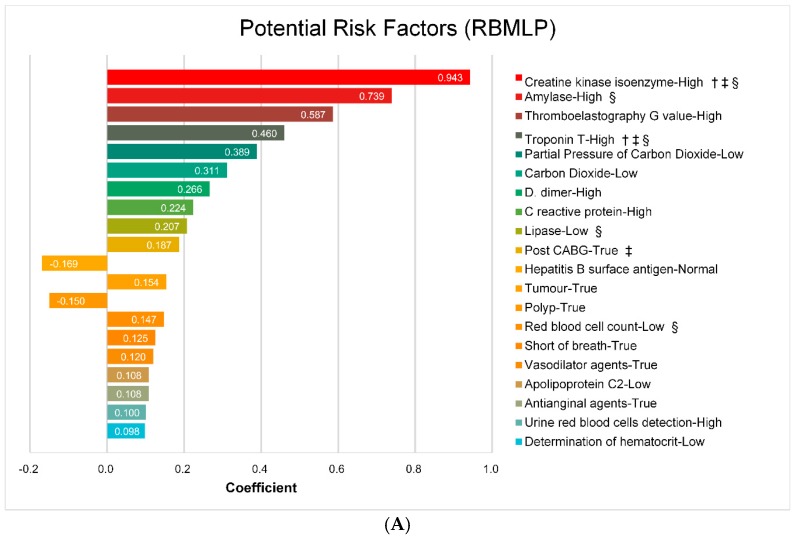

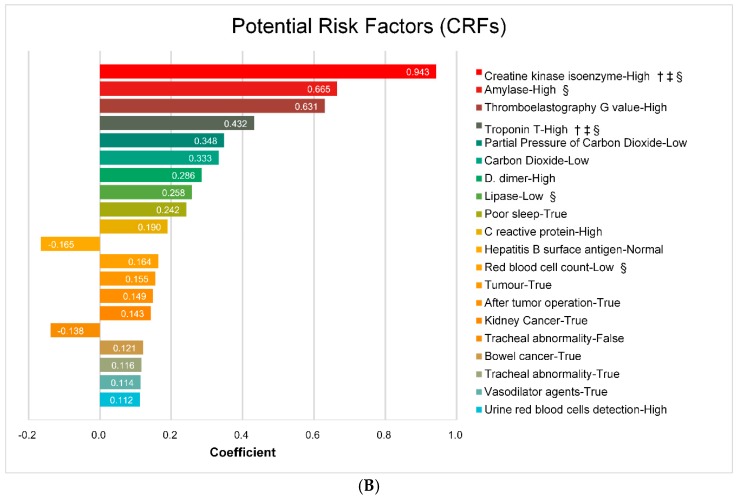
Potential risk factors based on ℓ1-LR model. (**A**) Risk factors based on RBMLP; (**B**) Risk factors based on CRFs (†: risk factor employed in GRACE; ‡: risk factor employed in TIMI; §: risk factor employed in top 20 of both random forest and ℓ1-LR model).

As illustrated in above section, all four learned models achieved competitive performances in MACE prediction. Among all these models, random forest achieved the best performance, which can offer the most powerful risk factors in predicting MACE. Therefore, we extract some important features from random forest models in terms of the mean decrease accuracy. Figure 6 shows the top-rank 20 risk factors in the random forest model. In addition, ℓ1-LR can extract some important features that influence the prediction results when the other models lack the interpretability [39]. Note that the positive value of the coefficient in ℓ1-LR model indicates an increasing possibility for occurrence of MACE, while the negative value means the opposite, in which the possibility is decreasing. Figure 7 shows the top 20 risk factors with their coefficients. 

As illustrated in Figure 6 and Figure 7, several risk factors show high discrimination abilities for ACS patient, which have been demonstrated in [6,7,40,41,42], such as *Age*, *Creatine kinase isoenzyme* and *Troponin T* as cardiac markers, *D-Dimer*, *C reactive protein* and *pro-brain natriuretic peptide* as biomarkers, *Killip class* and *Post CABG*. And also, some risk factors like *Operation for cancer*, *Short of breath* and medicine history, i.e., *Vasodilator* and *Antianginal agents*, also took an important role for MACE prediction. Moreover, *Hepatitis B surface Antigen-Normal* reduces the risk of MACE which obtains a complementary results with [13]. By comparing Figure 6 and Figure 7, we notice that several uppermost risk factors are both employed in both the GRACE and TIMI risk score models and mainly cardiac markers, which illustrates that our learned models are consistent with the traditional risk scores to some extent. Moreover, some risk factors overlapped in random forest and ℓ1-LR models, which indicates the machine learning algorithms can both discover the most discriminatory features from our data. Depending on the evaluation of clinical experts, all the extracted risk factors are indeed coherent and informative.

## 5. Discussion and Future Work

The ability of analyzing a large volume of EHRs in modern healthcare systems plays a vital role in the improvement of the quality of care delivery [43]. In this paper, we present a hybrid approach to utilize a specific type of EHRs, i.e., admission records, for MACE prediction. We first extracted features from admission records to describe patient condition at the early stage of their hospitalization. And then, the state-of-the-art supervised machine learning algorithms were employed to build our models for MACE prediction. In contrast to most traditional risk prediction approaches that require a well-controlled patient cohort to train the model with hand-picked risk factors, our approach automatically extracted patient information from unstructured admission records to establish MACE prediction models, extracted hidden risk factors from data, and has potential to provide nearly run-time decision support services for cardiologists in their daily work.

Experiments have been conducted on a real ACS dataset collected from a Chinese hospital to evaluate the performance of our approach. The experimental results indicate that our approach has a competitive generality with datasets extracted by different patient information extraction methods, and can achieve a comparative performance for MACE prediction. In addition, our models achieved significant improvements in comparison with both the GRACE and TIMI risk score models. Furthermore, the proposed approach can extract extra potential ACS risk factors according to ℓ1-LR model. It has been evaluated by clinical experts and the extracted risk factors are indeed coherent and informative.

It should be mentioned that there exist some limitations in our current work. First, although both feature extraction methods can sufficiently and efficiently extract patient features from the free text of admission records, we should notice that there exist some mistakes in the feature extraction results, which introduced interferences into the accurate descriptions of patient conditions. With regard to the rule-based model, it is necessary to extend/improve the lexicon and the rule set to reduce mistakes. However, it inevitably decreases the generalization of the model such that the over-fitting problem may come out. With regard to the CRFs model, the performance can be improved by considering more features for templates, such as Part-Of-Speech tags and Number tags, etc. and more training samples.

In addition, there are still several steps involving manual work in our rule-based and CRFs methods, such as rule definition and CRFs training set annotation. These steps are essential and crucial for the feature extraction but time-consuming work needed professional knowledge, which limits our proposed method’s scalability. The limitation of scalability in rule-based method is an everlasting problem since it was proposed, but it is a very useful method because of its remarkable performance for specific environment. On the other hand, the machine learning method, i.e., CRFs, can obtain the better performance with more varied and comprehensive training set, which means that it can become more and more powerful along with the increase of training samples. Moreover, to prove the concept in our study, we manually annotated the MACE occurred in patients’ progress notes. However, it is a tedious and time-consuming process for human evaluation on a large volume of patient samples. To this end, semi-supervised learning could provide a feasible solution since it can use readily available unlabeled data to improve supervised learning tasks when the labeled data are scarce or expensive [39]. The annotated data, on the other hand, provides a valuable benchmark for future semi-supervised learning technology development for us. Moreover, we should notice that different definitions of MACE as composite end points may lead to a different experiment results and conclusions [4], so we need to explore the influences of different definitions of MACE on the prediction performances in our future work.

## 6. Conclusions

In this paper, we have presented a hybrid approach to utilize the admission records for MACE prediction of ACS patients. The experimental results indicate that using admission records can effectively provide MACE prediction services for ACS patients at the early stage of their hospitalizations. As for future work, we intend to carry out a large scale of experiments and evaluate the model performance on a larger scale of EHRs of ACS patients, as a crucial advantage over traditional techniques for MACE prediction.

## Figures and Tables

**Table 1:** Four rules used for matching clauses.

Rule Expression	Regular Expression of Rule	Matched Clause (English Translation) and Annotated Clause
[Body Part] + [Symptom]	(?<BPPatternSet>(BP#[0–9]+#)+)[\u4e00–\u9fa5]*(?<STDGPatternSet>(ST#[0–9]+#)+)	有双下肢水肿 (Having lower extremity edema) 有BP#2#ST#1#
[Body Part] + [Description]	(?<BPPatternSet>(((BP#[0–9]+#)\*)+)[\u4e00–\u9fa5]*(?<DSPattern>DS#[0–9]+#)	行冠状动脉CT检查提示前降支、回旋支弥漫性狭窄 (The result of coronary CT showed there were diffuse stenosis in anterior descending artery and circumflex artery.) 行BP#5#EX#4#检查提示BP#3#\BP#2#弥漫性DS#1#
[Diagnosis]	(?<DGPattern>(DG#[0–9]+#))	无明显诱因出现胸闷 (Feeling chest tightness without any obvious incentive) 无明显诱因出现DG#1#
[Medication]	(?<MDPatternSet>MD#[0–9]+#)	术后口服“阿司匹林”等药物 (Oral drugs including Aspirin after operation) 术后口服“MD#1#”等药物

**Table 2:** Conditional random fields feature template.

No.	Feature	*n* Value
1	C_n_	*n* = −3, −2, −1, 0, 1, 2, 3
2	C_n_/C_n+1_	*n* = −3, −2, −1, 0, 1, 2
3	P(C_n_)	*n* = −3, −2, −1, 0, 1, 2, 3
4	N(C_n_)	*n* = −3, −2, −1, 0, 1, 2, 3

**Table 3:** The summary statistics of pool of patient samples.

Item	All Patient Samples	Controls	Patient Samples with MACE
Patient Samples	2930	2178	752
Male (%)	2080 (70.99%)	1553 (71.30%)	527 (70.08%)
Age (S.D.)	62.27 (±12.11)	60.60 (±11.71)	67.13 (±11.95)
Average LOS (S.D.)	8.20 (±7.07)	5.94 (±3.01)	14.77 (±10.51)

**Table 4:** The summary statistics of feature categories.

Method	Feature Categories (*: Free Text Features)								
	Diagnosis-Related *	Symptom-Related *	Medicine-Related *	Surgery-Related *	ECG-Related *	Personal-Related *	Vital Signs-Related	Lab Test-Related	TotalNumber
**RBMLP**	49	25	9	7	8	7	7	156	268
**CRFs**	56	26	11	15	6	7	7	156	284

**Table 5:** The summary statistics of features.

Vital Signs-Related Features	Mean	S.D.	Lab Test-Related Features	Frequency	RBMLP Free-Text Features	Frequency	CRFs Free-Text Features	Frequency
BT (°C)	36.2	0.3	HDL-C	1273	CHD	2865	CHD	2841
Pulse (bpm)	73.4	9.7	Calcium	994	Angina	2787	UA	2354
BR (bpm)	18.1	0.5	Triglycerides	991	Arteriosclerosis	2267	Arteriosclerosis	2046
SBP (mmHg)	132.1	17.6	Glucose	867	CPS	2149	Hypertension	1990
DBP (mmHg)	77.6	10.2	Neutrophils	852	Hypertension	1988	CPS	1826
Height (cm)	167.0	8.1	Urine color	831	PRP	1561	PRP	1573
Weight (kg)	71.8	12.4	Eosinophils	818	Anti-H therapy	1375	Postoperative	1372
			Lymphocytes	773	Anti-A therapy	1216	Anti-H therapy	1361
			Monocytes	633	Anti-C therapy	1216	Anti-C therapy	1188
			Apo A-1	546	Sweating	1115	Anti-A therapy	1178
					Smoking	1115		

BT: body temperature; BR: breathe rate; SBP: systolic blood pressure; DBP: diastolic blood pressure; HDL-C: high density lipoprotein-cholesterol; Apo A-1: apolipoprotein A-1; CHD: Coronary Heart Disease; CPS: chest pain and stuffiness; PRP: precordial region pain; Anti-H therapy: anti-hypertension therapy; Anti-A therapy: anti-angina therapy; Anti-C therapy: anticoagulant therapy; UA: unstable angina.

**Table 6:** The AUC values using 5-fold cross validation (*: 95% CI by 10 times repetition of the learning process).

Methods	SVM *		RF *		NB *		ℓ_1_-LR *		GRACE	TIMI
	AUC	CI	AUC	CI	AUC	CI	AUC	CI	AUC	AUC
**RBMLP**	0.703	0.701–0.705	0.724	0.722–0.725	0.695	0.693–0.696	0.705	0.702–0.708	0.636	0.579
**CRFs**	0.705	0.703–0.708	0.723	0.722–0.724	0.695	0.694–0.697	0.706	0.704–0.709	0.641	0.576

**Table 7:** Statistical differences between all learned models and the baseline models.

Model		RBMLP						CRFs						Row
		RF	SVM	NB	ℓ1-LR	GRACE	TIMI	RF	SVM	NB	ℓ1-LR	GRACE	TIMI	
**RBMLP**	RF	1 •	0.0015 **	6.81 × 10^−5^ ***	0.0034 **	3.21 × 10^−15^ ***	2.2 × 10^−16^ ***	0.6636 •	0.0050 **	8.67 × 10^−5^ ***	0.0083 **	8.32 × 10^−14^ ***	2.2 × 10^−16^ ***	1
	SVM		1 •	0.2286 •	0.9735 •	1.19 × 10^−8^ ***	2.2 × 10^−16^ ***	0.0028 **	0.4013 •	0.249 •	0.8166 •	1.18 × 10^−8^ ***	2.2 × 10^−16^ ***	2
	NB			1 •	0.1847 •	1.77 × 10^−6^ ***	2.2 × 10^−16^ ***	0.0001 ***	0.1511 •	0.7042 •	0.1189 •	1.22 × 10^−5^ ***	2.2 × 10^−16^ ***	3
	ℓ1-LR`				1 •	1.58 × 10^−11^ ***	2.2 × 10^−16^ ***	0.0056 **	0.7382 •	0.2031 •	0.4196 •	4.28 × 10^−10^ ***	2.2 × 10^−16^ ***	4
	GRACE					1 •	8.89 × 10^−6^ ***	5.29 × 10^−15^ ***	3.62 × 10^−9^ ***	1.31 × 10^−6^ ***	1.40 × 10^−12^ ***	0.0159 •	4.03 × 10^−6^ ***	5
	TIMI						1 •	2.2 × 10^−16^ **	2.2 × 10^−16^ ***	2.2 × 10^−16^ ***	2.2 × 10^−16^ ***	1.28 × 10^−6^ ***	0.5885 •	6
**CRFs**	RF							1 •	0.0062 **	0.0001 ***	0.0106 •	1.31 × 10^−13^ ***	2.2 × 10^−16^ ***	7
	SVM								1 •	0.1584 •	0.9442 •	3.88 × 10^−8^ ***	2.2 × 10^−16^ ***	8
	NB									1 •	0.1261 •	9.32 × 10^−6^ ***	2.2 × 10^−16^ ***	9
	ℓ1-LR										1 •	4.70 × 10^−11^ ***	2.2 × 10^−16^ ***	10
	GRACE											1 •	4.33 × 10^−7^ ***	11
	TIMI												1 •	12
	Column	A	B	C	D	E	F	G	H	I	J	K	L	

***: *p*-value < 0.005; **: *p*-value < 0.01; *: *p*-value < 0.05; •: *p*-value > 0.05.

**Table 8:** The AUC values of models-GRACE using 5-fold cross validation (*: 95% CI by 10 Times repetition of learning process).

Methods	SVM-GRACE *		RF-GRACE *		NB-GRACE *		ℓ_1_-LR-GRACE *	
	AUC	CI	AUC	CI	AUC	CI	AUC	CI
RBMLP	0.622	0.610–0.634	0.687	0.685–0.689	0.692	0.691–0.693	0.698	0.697–0.699
CRFs	0.620	0.608–0.632	0.683	0.681–0.686	0.691	0.690–0.692	0.697	0.696–0.698

**Table 9:** Statistical differences between models-GRACE and GRACE, all learned models.

Methods		SVM-GRACE	RF-GRACE	NB-GRACE	ℓ_1_-LR-GRACE
RBMLP	GRACE	0.1827 •	3.45 × 10^−6^ ***	1.7 × 10^−7^ ***	6.2 × 10^−10^ ***
	Learned models	5.55 × 10^−12^ ***	2.05 × 10^−5^ ***	0.6802 •	0.1098 •
CRFs	GRACE	0.05384 •	1.54 × 10^−4^ ***	3.72 × 10^−6^ ***	8.01 × 10^−8^ ***
	Learned models	2.47 × 10^−13^ ***	3.86 × 10^−6^ ***	0.5267 •	0.02104 *

*** *p*-value < 0.005; ** *p*-value < 0.01; * *p*-value < 0.05; • *p*-value > 0.05.
